# Projecting influenza vaccine effectiveness: A simulation study

**DOI:** 10.1371/journal.pone.0241549

**Published:** 2020-11-03

**Authors:** Thomas N. Vilches, Affan Shoukat, Claudia Pio Ferreira, Seyed M. Moghadas

**Affiliations:** 1 Institute of Mathematics, Statistics and Scientific Computing, University of Campinas, Campinas, SP, Brazil; 2 Center for Infectious Disease Modeling and Analysis, School of Public Health, Yale University, New Haven, CT, United States of America; 3 Department of Biostatistics, Institute of Biosciences, São Paulo State University, Botucatu, SP, Brazil; 4 Agent-Based Modelling Laboratory, York University, Toronto, Ontario, Canada; University of Georgia, UNITED STATES

## Abstract

The impact of influenza vaccination is largely measured by estimating vaccine effectiveness (VE), which vary in different seasons. Strain mutations and waning immunity present two key mechanisms affecting VE. We sought to quantify the relative effect of these mechanisms by projecting VE and the reduction of illness due to vaccination. We developed a stochastic age-structured agent-based simulation model of influenza transmission dynamics to encapsulate intraseason waning of immunity post-vaccination, and mutation-induced antigenic distance between circulating strains and vaccine strains. Parameterizing the model with published estimates, we projected the temporal and overall VE during an epidemic season, and estimated the reduction of infection for high (70%) and low (30%) vaccine efficacies to reflect the levels of vaccine-induced protection in randomized control trials. Both temporal and overall VE decreased as the attack rate increased, with lower median values for epidemics starting with strains that were antigenically more distant from vaccine strains. We observed a higher rate of temporal decline with considerably lower median values of the overall VE in the presence of intraseason waning of immunity compared with only the antigenic distance effect. The highest benefit of vaccination in preventing influenza infection was achieved at moderate attack rates in the range of 6%-15%. The results show that even when VE is relatively low in the population and almost negligible for older age groups (i.e., 50+ years), vaccination can still prevent significant illness in high-risk individuals; thereby reducing healthcare resource utilization and economic burden. Our study indicates that early vaccination remains an important strategy for alleviating the burden of seasonal influenza. Policy discussions on optimal timing of vaccination to reduce the effect of intraseason waning of immunity should be considered in the context of strain mutations within the epidemic course.

## Introduction

Seasonal vaccination is the most effective preventive measure against influenza infection and its severe outcomes such as hospitalization and death [1, 2]. There are two important properties that quantify the impact of influenza vaccination. First is the vaccine efficacy in infection prevention, which depends on several factors at the individual level such as health status (e.g., the presence of comorbid conditions), age, and frailty that impairs the ability to resist infection and respond to vaccination in a process known as immunosenescence [3–7]. Second is the vaccine effectiveness (VE), which depends not only on vaccine efficacy, but also on other population and pathogen characteristics including the occurrence of strain mutations during natural infection that enhances the antigenic distance between dominant influenza viruses and the vaccine strains [8–10].

While the vaccine efficacy is measured in clinical trials (under ideal conditions), the overall VE is estimated during an influenza season (under real-world conditions). Both measures estimate how well the vaccine performs to prevent infection, but they are subject to the aforementioned factors in their estimates. Observational studies indicate that VE often declines as the epidemic progresses and infection spreads through the population [11, 12]. Explicators for the decline of VE have included host factors, suboptimal immunity, attack rates, and viral drift that reduces vaccine-induced protection. Further, recent studies provide evidence of intraseason waning immunity (hereinafter referred to as waning immunity) among individuals vaccinated with the traditional inactivated vaccine [13–16]. These studies measure effectiveness using methods of “test-negative design” [12, 17, 18] and respiratory syncytial virus illness as a “negative-control outcome” [14], without consideration of infecting strains and their potential antigenic distance with vaccine strains. Thus, the relative impact of waning vaccine-induced protection and viral drift on reducing VE during a single epidemic season remains undetermined.

We sought to quantify the impact of two distinct mechanisms of antigenic distance and waning immunity on the reduction of VE, by developing an agent-based multi-scale simulation model of influenza transmission dynamics in a population. We parameterised the model with published estimates and simulated it to project the population-wide benefits of vaccination for different scenarios of disease severity (i.e., reflected in epidemic attack rate), vaccine efficacy in infection prevention (i.e., high and low), and the presence of virus strains of varying antigenic distance at the onset of epidemic. We compared the model outcomes under the scenarios of antigenic distance and waning immunity in terms of the overall and temporal VE, and the percentage reduction of illness achieved due to vaccination.

## Materials and methods

### Ethics statement

This study used publicly available data and information published in previous studies, and therefore did not require ethics approval. No patient records were used in this study.

### The model framework

We developed a stochastic age-structured agent-based simulation model of influenza transmission dynamics, which includes health statuses of individuals as unvaccinated, vaccinated, latent (exposed and infected), symptomatically infectious, asymptomatically infectious, and recovered (S1 Fig in S1 Appendix). In an epidemic scenario, we assumed that recovery from infection confers immunity against re-infection in the same season.

### Population demographics and contact patterns

We stratified the population into 5-year age groups in a population of 10,000 individuals. The age groups were populated according to the distribution (S2 Fig in S1 Appendix) derived from the 2017 Canadian census data [19]. The age of each individual was randomly selected in the associated 5-year age-range. Due to short timelines of a single influenza epidemic season, we ignored demographics of birth and natural death. We assigned a frailty index to each individual by performing a segmented linear regression (S1 Fig and S1 Table in S1 Appendix) on the 2016 Canadian Community Health Survey data of chronic diseases [20]. This regression, as a function of age, was used to sample frailty index from a uniform distribution, with the mean given by the value on the linear regression at any given age and a standard deviation of 5% [21]. The daily number of contacts for each individual was sampled from a negative-binomial distribution [22], with age-dependent mean and standard deviations (S2 Fig, S2 Table in S1 Appendix).

### Strain mutation and antigenic distance

We considered hemagglutinin sequences with lengths of 566 amino acids in a virus strain, herein referred to as ‘sites’ [10]. We calculated the probability of mutation in each site of a strain sequence [9], taking into account possible substitutions reflecting a 20-dimensional immunological shape space [23], by:

$$P_{mutation}=1-e^{\frac{-\mu T}{365}}$$

where μ is the rate of nucleotide substitution/site/year and *T* is the infectious period (days) of the individual infected with the same strain. Each site of a strain sequence was associated with 20 possible substitutions. When a site was selected to change, the day on which the substitution occurred was randomly chosen within the first half of the infectious period of an infected individual for the mutant to emerge. We allowed for more than one change in sites of a single strain. This process was applied to all strains in an infected individual. The antigenic distance, denoted by *d*, between a circulating strain and the vaccine strains was then calculated by the ratio of the number of substitutions in sequence sites to the total number of sites [8, 9]. We used this distance to determine the strain-specific vaccine efficacy in infection prevention described below.

### Vaccination and vaccine efficacy

Individuals were vaccinated randomly according to the vaccine coverage estimates (S3 Table in S1 Appendix) provided by the Public Health Agency of Canada for the 2016/17 seasonal influenza in different age groups [24]. For vaccine distribution, we changed the status for a proportion of individuals from susceptible to vaccinated corresponding to the vaccine coverage in their age group at the onset of model simulations. We then considered the initial vaccine efficacy *V_e_* (as model input) to reflect the level of infection prevention in randomized control trials under ideal conditions. For the model scenario with virus mutations, the protection level of a vaccinated individual against infection with a strain was determined by the sampled frailty index and the strain antigenic distance. The strain-specific protection conferred by vaccination was then calculated by *E_s_* = *V_e_*(1−*f*)−7.37*d*, where *f* is the sampled frailty index of a vaccinated individual. The slope of decline was parameterized based on linear regression of VE for ILI correlates in several influenza seasons from 1982 to 2008 as a decreasing function of antigenic distance [9]. In the model scenario with waning immunity (without considering virus mutations), we calibrated the model to the estimated 7% absolute decline of VE per month post-vaccination [13] to determine the temporal reduction of vaccine efficacy *E_t_* at the individual level. Vaccine-induced protection was included as a reduction factor for disease transmission when a vaccinated individual encountered an infectious individual. If infection occurred, vaccine protection reduced the probability of developing symptomatic disease by a factor of 1−*E_s_* or 1−*E_t_* in the corresponding model scenarios of antigenic distance and waning immunity.

### Disease dynamics

We allowed the disease to spread between individuals, in a probabilistic approach, through contacts with symptomatically or asymptomatically infectious individuals. This was implemented in the model as rejection sampling-based (Bernoulli) trials where the chance of success is defined by a transmission probability distribution. Based on the natural history of influenza (S1 Fig in S1 Appendix), a newly infected individual entered a latent stage during which the disease cannot be transmitted. Once the latent period elapsed, the disease was manifested as either symptomatic (with clinical symptoms) or asymptomatic (without clinical symptoms). Latent and infectious periods were sampled for each individual from their associated distributions described in the Parameterization Section below. For a successful transmission in the antigenic distance model at the time of contact, we determined the infecting strain using the roulette fitness proportionate selection (*α_i_*) [25], given by:

$$\alpha_{i}=\frac{(1-E_{i}){\Delta t}_{i}}{\sum_{j}(1-E_{j}){\Delta t}_{j}}$$

where *E_i_* is the vaccine protection efficacy against mutant *i*, and Δ*t_i_* is the time since emergence of mutant *i* during the infectious period.

### Parameterization

The baseline transmission probability in the absence of vaccination was obtained by calibrating the model to various attack rates, defined here as the percentage of an unvaccinated population infected throughout a season. In a recent systematic review [26], the pooled estimates for symptomatic and asymptomatic infections combined for all influenza (A and B) among unvaccinated individuals were 22.5% (95%CI 9.0% - 46.0%) for children (<18 years) and 10.7% (95%CI 4.5% - 23.2%) for adults. We considered a range of attack rates from low (4%) to high (40%) for calibration in the absence of vaccination, representing mild to severe influenza epidemic seasons.

Latent period was drawn from a uniform distribution with the mean of 1.5 days within the range of 1–2 days [27, 28]. The infectious periods for both symptomatic and asymptomatic infections were sampled from a truncated lognormal distribution with scale of 1 and shape of 0.4356, having mean of 3.38 days [27, 28]. The probability of developing asymptomatic infection was sampled for each individual from a uniform distribution in the range 0.3–0.7 and was modified for vaccinated individuals according to the vaccine protection. We considered two vaccine efficacies of 70% (high) and 30% (low), from which the individual-level vaccine protection efficacy was calculated based on the sampled frailty. All parameters and associated ranges are summarized in Table 1.

**Table 1:** Model parameters with their associated values (ranges).

Parameter	Value/range	Source/comment
Attack rate in the absence of vaccination	4%–40%	[26]
Baseline transmission probability	Varied depending on the attack rate	Calibrated to a specific attack rate
Mean latent period (days)	1.5; Uniform(1, 2)	[27, 28]
Mean infectious period (days)	3.38; LogNormal(1, 0.4356)	[27, 28]
Relative transmissibility of asymptomatic infection	0.3–0.7	[27, 28] Sampled for each individual
antigenic distance at the onset of epidemic	0–0.04	[8, 9]
Annual strain mutation rate	0.004–0.31, Varied depending on the attack rate	[8, 9] Calibrated to the estimated antigenic distance
Waning vaccine-induced protection at the individual level	1.6% per week	[13] Calibrated to 7% decline of VE per month
Vaccine efficacy	70% (high); 30% (low)	Assumed
Frailty	Sampled	[20] Varied depending on the age of individuals

### Model implementation and estimation of VE

The model was computationally developed in Julia language to perform Monte-Carlo simulations. Vaccination was implemented in the model as pre-epidemic program. All simulations were seeded with a randomly selected individual in the latent state of the disease. We ran 3000 independent realizations for each scenario, and used five age groups (0.5–8, 9–17, 18–49, 50–64, and 65+ years) to aggregate the simulation outputs for disease incidence and estimate the overall and age-specific VE. Incidence of infection during the epidemic and any change in the health status of individuals were recorded. We estimated vaccine effectiveness using *VE* = (1−*OR*)100%, where *OR* is the odds ratio for cumulative incidence among vaccinated versus unvaccinated individuals [29].

### Simulation scenarios

For the antigenic distance model, we considered five scenarios in which the circulating strains were introduced with different antigenic distances in the range 0–0.04 [8, 9] at the onset of epidemic. We then tracked the emerging strains in infected individuals to project the trend of maximum antigenic distance throughout the epidemic (S3, S4 Figs in S1 Appendix). For the waning immunity model, the vaccine protection efficacy at the individual level was reduced every week by the calibrated value (Table 1) to achieve 7% absolute reduction of VE per month post-vaccination [13]. Model outcomes for different scenarios were analysed, including the incidence of infection and the temporal change in VE.

## Results

We analysed the outcomes of the scenarios described above in terms of temporal antigenic distance and incidence of disease during an outbreak for a given attack rate. In the antigenic distance model, the results indicate an increase (S3, S4 Figs in S1 Appendix) in the antigenic distance within the range estimated using a sequence-based method for H1N1 strains during several epidemic seasons [8, 9]. The incidence of disease was higher for epidemics starting with strains that carry a higher antigenic distance compared with vaccine strains (S5-S7 Figs in S1 Appendix).

### Overall VE

**Fig 1:**
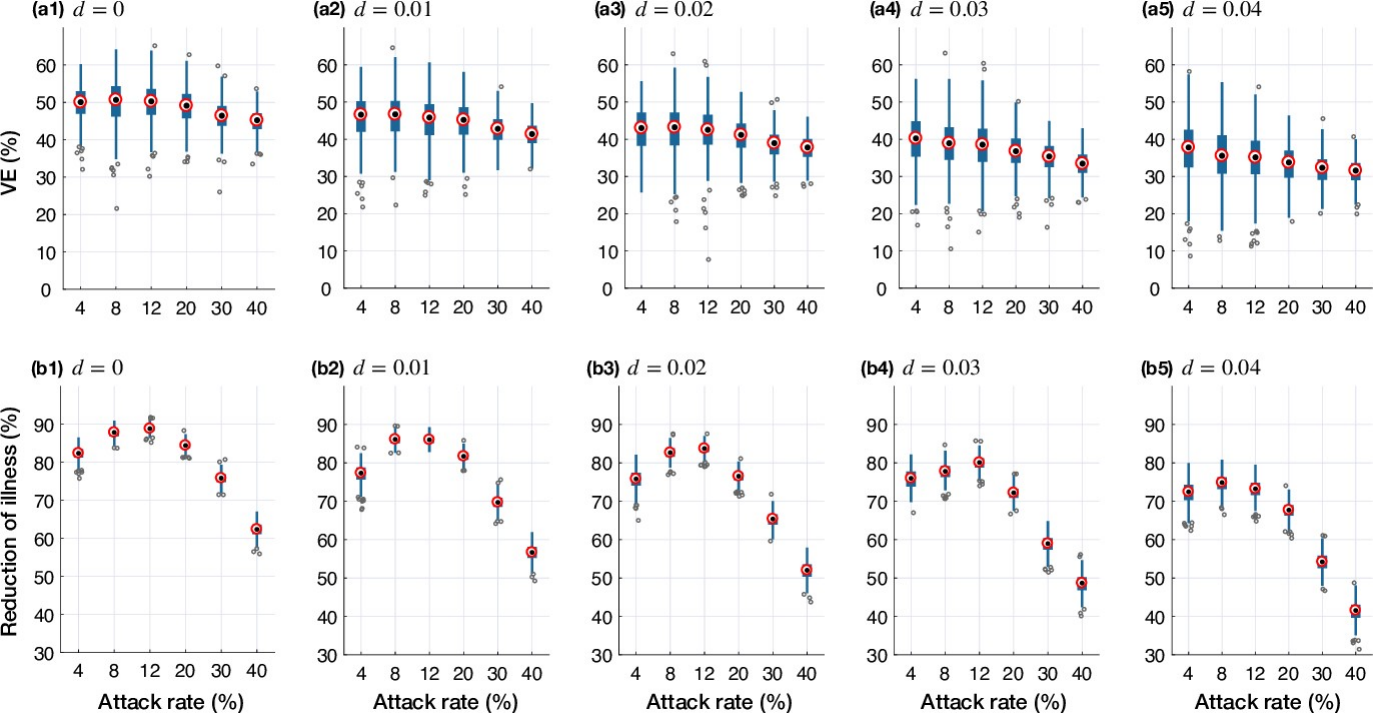
Projected VE (a1-a5) and reduction of illness (b1-b5) in the antigenic distance model with an initial vaccine efficacy of 70% for calibrated attack rates in the absence of vaccination. Simulated scenarios correspond to the presence of circulating strains with different antigenic distance at the onset of epidemic. Boxplots represents the variation in projected VE and reduction of illness with median values shown by red circles.

For a vaccine efficacy of 70% in infection prevention, Fig 1 (a1-a5) shows the overall VE for different attack rates in the antigenic distance model. Not surprisingly, VE decreases as the epidemic attack rate increases with a lower median VE for epidemics starting with strains that have a higher antigenic distance. However, despite decreasing VE, we observed that the highest reduction of illness is achieved for some moderate attack rates in the range 6%-15%. Performing a segmented linear regression suggests that the median reduction of illness increases for low to moderate (4%-12%) attack rates and decreases for higher attack rates Fig 1 (b1-b5). A higher initial antigenic distance was associated with lower estimates of reduction of illness, decreasing the benefits of vaccination without changing the observed pattern.

**Fig 2:**
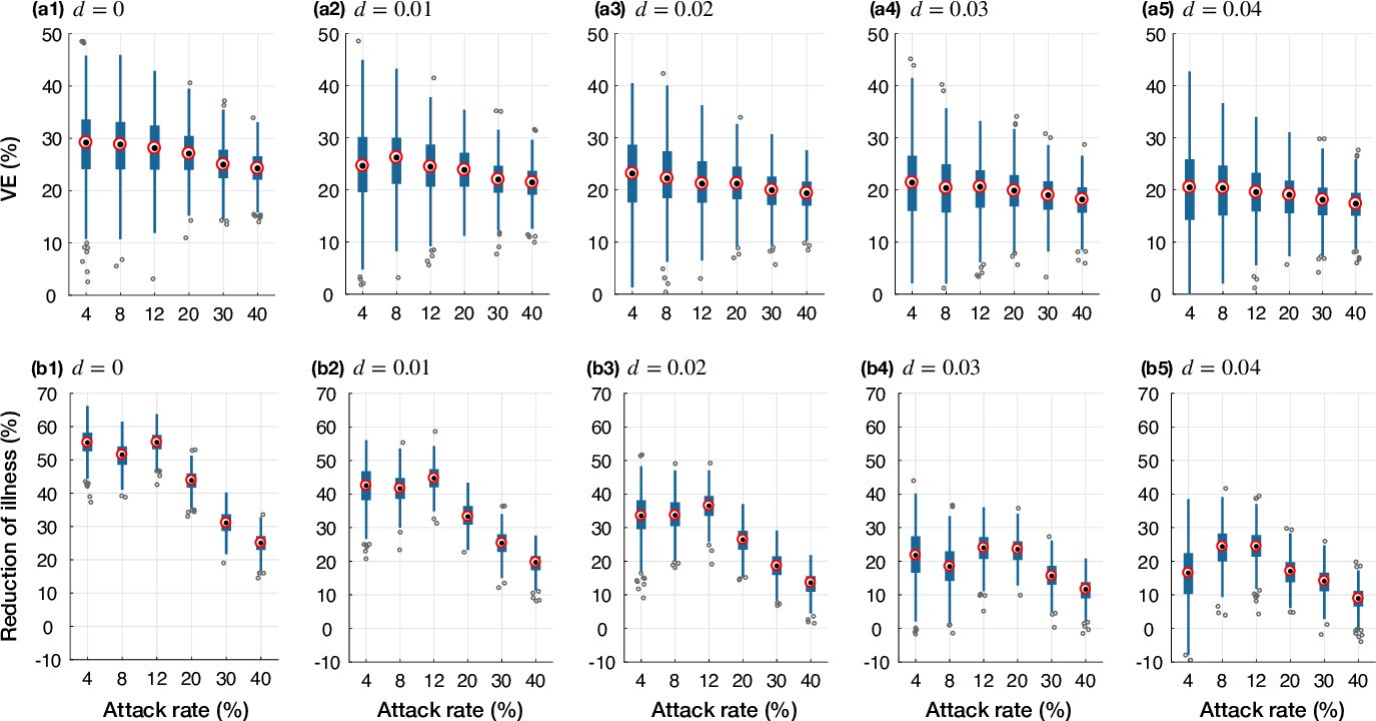
Projected VE (a1-a5) and reduction of illness (b1-b5) in the antigenic distance model with an initial vaccine efficacy of 30% for calibrated attack rates in the absence of vaccination. Simulated scenarios correspond to the presence of circulating strains with different antigenic distance at the onset of epidemic. Boxplots represents the variation in projected VE and reduction of illness with median values shown by red circles.

**Fig 3:**
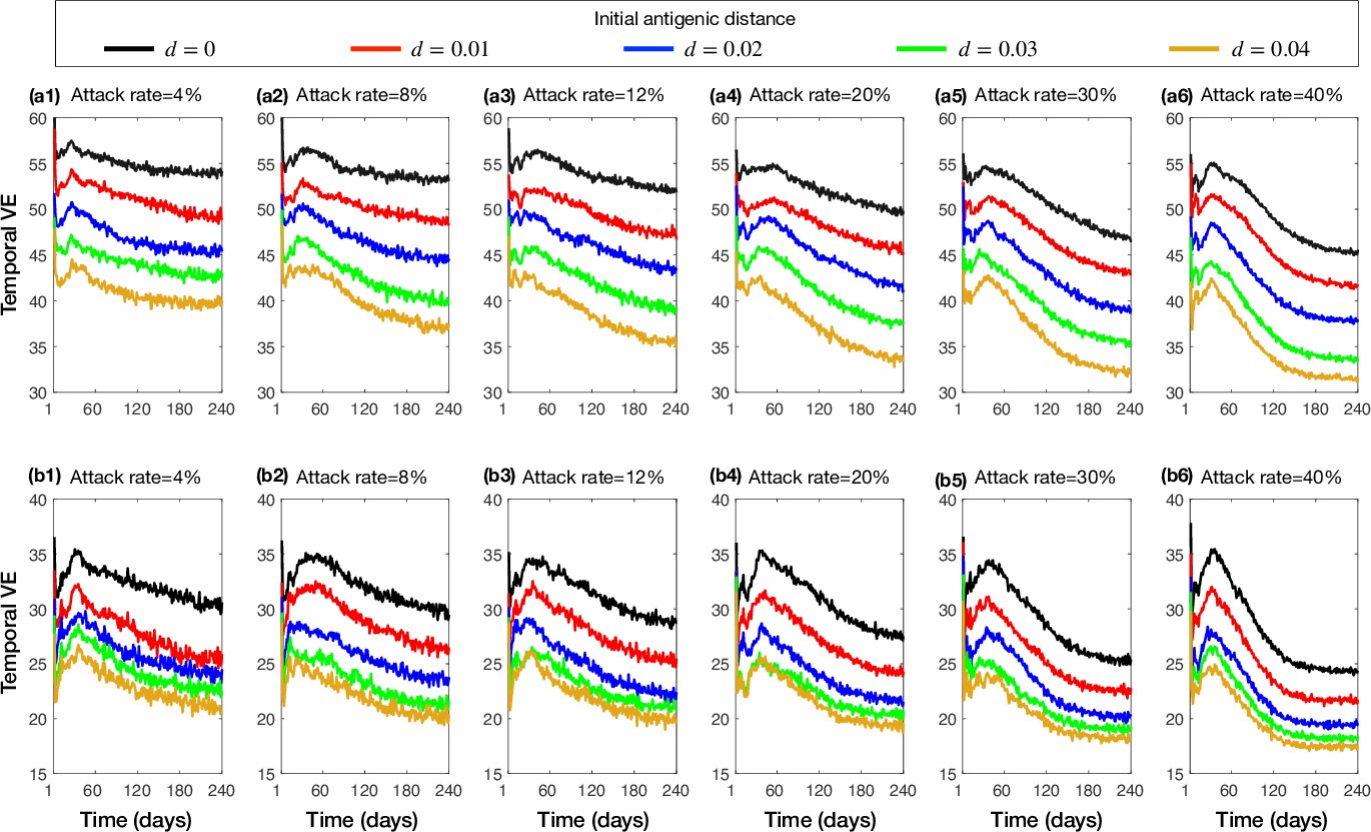
Temporal median VE in the antigenic distance model with calibrated attack rates in the absence of vaccination. Colour curves correspond to epidemics starting with strains of different antigenic distances in the range 0–0.04. Vaccine efficacy was set to 70% (a1-a6) and 30% (b1-b6).

The qualitative aspects of these results did not alter when the epidemic scenarios were simulated with a low vaccine efficacy of 30% (Fig 2). As expected, the temporal VE declined throughout the simulated epidemics with both low and high vaccine efficacies (Fig 3), corresponding to increasing antigenic distance patterns (S3, S4 Figs in S1 Appendix).

**Fig 4:**
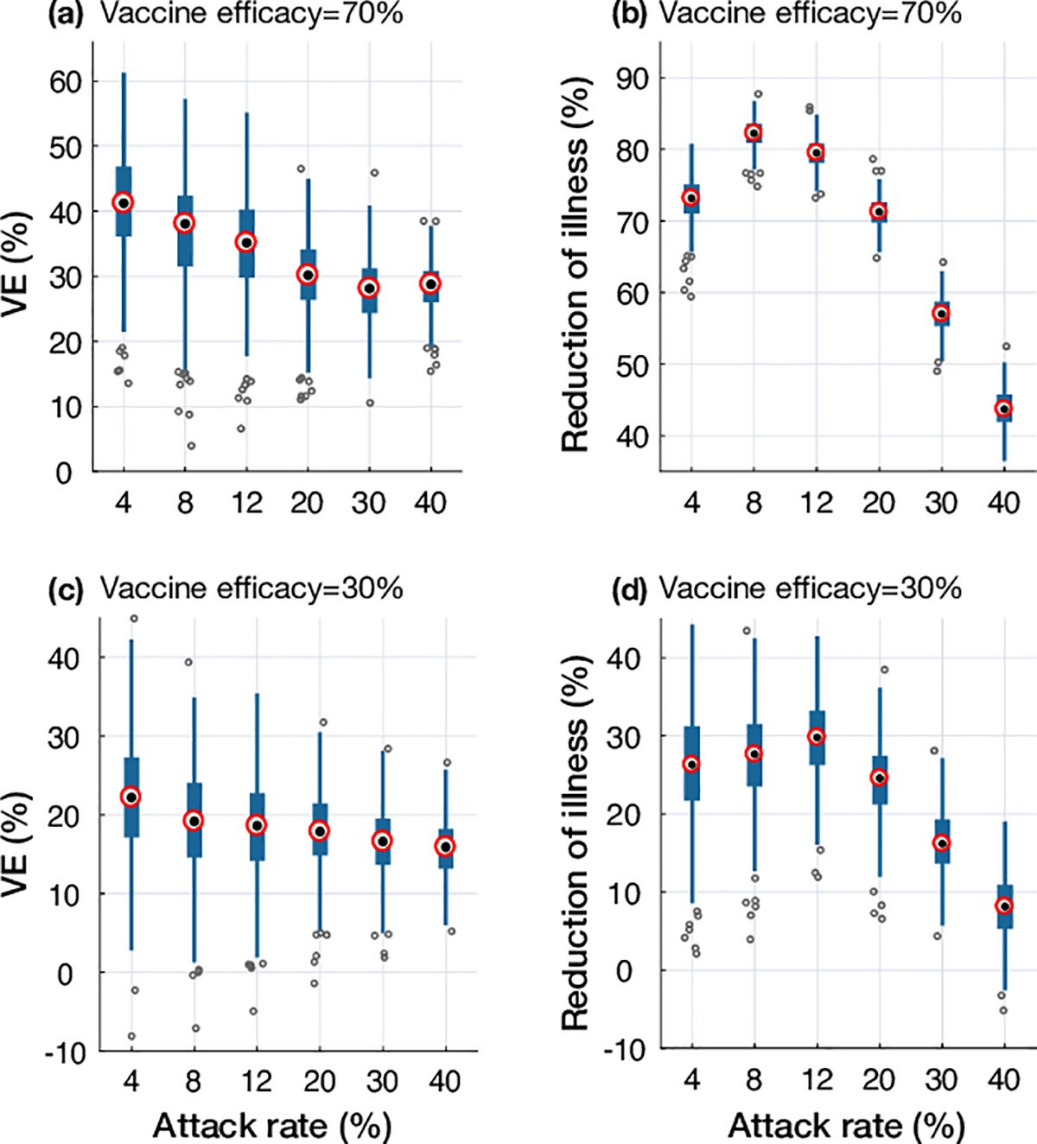
Projected VE and reduction of illness in the waning immunity model with an initial vaccine efficacy of 70% (a,b) and 30% (c,d) for calibrated attack rates in the absence of vaccination. Boxplots represent the variation in projected VE and reduction of illness with median values shown by red circles.

**Fig 5:**
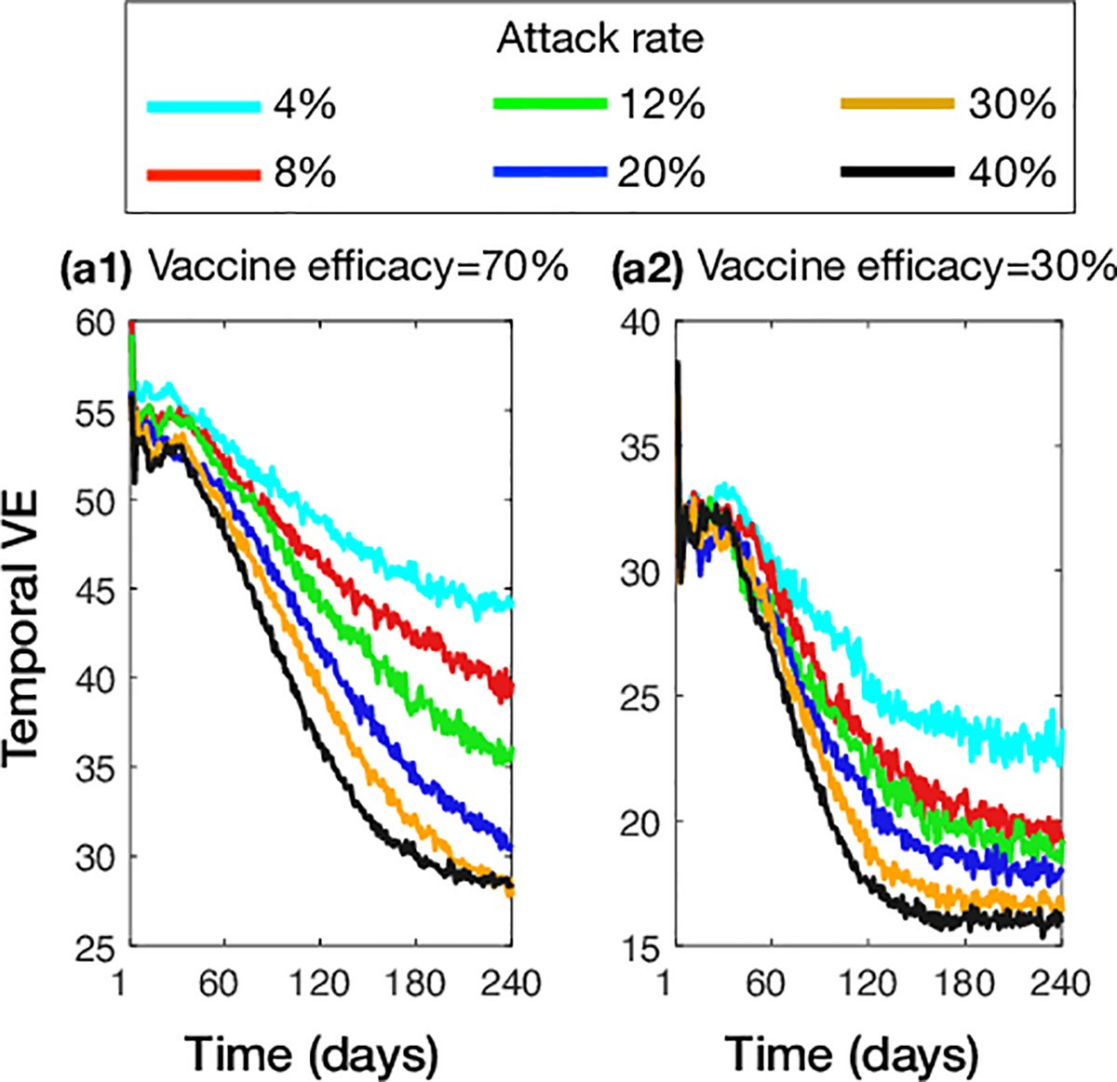
Temporal median VE in the waning immunity model with calibrated attack rates in the absence of vaccination. Vaccine efficacy was set to 70% (a1), and 30% (a2).

We also observed similar outcomes when simulating the waning immunity model with 70% and 30% vaccine efficacies (Fig 4). While VE decreased as the attack rate increased, the reduction of illness was highest for moderate attack rates (8%-12%) with lower vaccine benefits in low and high attack rates. Similar to the model of antigenic distance, the temporal VE in the waning immunity model declined throughout the simulated epidemics with both low and high vaccine efficacies (Fig 5) but saturated at a considerably lower levels (for the overall VE) compared the antigenic distance model.

### Age-specific VE

In all scenarios simulated here, the lowest VE was associated with age groups of 50+ years old, reflecting lower individual-level vaccine-induced protections due higher frailty compared to other age groups (S8, S9 Figs in S1 Appendix). However, the median reduction of illness in the 50+ years age-groups was comparable or higher than other age groups, even with negligible (nearly zero) median VE when the attack rate was relatively high, or vaccine efficacy was low (30%) (S10 Fig in S1 Appendix). This indicates the indirect benefits of vaccination that reduces the burden of illness in older individuals as a result of increased herd immunity.

## Discussion

The modelling outcomes in this study show that the highest benefit of vaccination in preventing influenza infection is achieved at moderate attack rates. This finding corroborates a recent data-driven study estimating VE during the 2017–2018 influenza season in the United States [30]. The study concludes that despite a relatively low estimated VE of 38% in the population (which also varied in different age groups), the benefit of influenza vaccination was substantial mainly because of the high burden of influenza-associated disease. In our model, this corresponds to a maximum reduction of illness observed for moderate attack rates, presenting an extremum for the effect of vaccination. For mild seasons with a low attack rate, the benefits of vaccination are largely masked by the low number of infections in the population. On the other hand, for severe seasons with a high attack rate, both the VE and reduction of illness can decrease substantially, diminishing the impact of vaccination as a result of high number of infections and the rapid spread of disease. However, our model shows that even when VE is relatively low in the population and almost negligible for older age groups (i.e., 50+ years), vaccination can still prevent significant illness in high-risk individuals; hence reducing healthcare resource utilization and economic burden.

Our model included two distinct processes of virus mutation and waning immunity. The fact that mutations occur, not only from one season to the next, but also during each season is an important factor determining the risk of infection in vaccinated individuals [7]. As new mutants evolve, the antigenic distance may be increased, which reduces the efficacy of vaccine in preventing infection [8, 9]. Within the published estimates, we observed that waning immunity can lead to a larger reduction of VE compared to only antigenic distance. Studies on intraseason waning immunity [12–14, 17, 18] raise the concern that early vaccination may leave vaccinated individuals, especially those in high-risk groups, vulnerable to infection during the epidemic peak, highlighting the importance of a policy discussion regarding the optimal timing of vaccination. Due to various factors affecting the onset of epidemic season and its severity [7, 31], determining this optimal timing poses a significant challenge. On one hand, later vaccination during the epidemic delays the effect of waning vaccine-induced protection, and therefore reduces vulnerability of vaccinated individuals during the high influenza activity. On the other hand, it allows for the epidemic to spread at a faster pace during the early stages, which can encourage strain mutants with high antigenic distance to emerge more quickly and diminish the vaccine-induced protection. Early vaccination can slow the spread of disease, and therefore decelerate the generation of mutants that are antigenically distant from those included in the vaccine. Since viral mutation and waning immunity occur concurrently in a season, a potential solution to limit the decline of VE would be to enhance the level of heard immunity by increasing the vaccination coverage, or to provide a booster dose which represents a costly strategy. Raising herd immunity requires strategies with improved vaccination coverage. Because the highest burden of influenza in terms of mortality and hospitalization occurs in older individuals and those with comorbid illnesses, vaccination programs have prioritized these high-risk individuals. Vaccination provides real but limited protection in these groups [32–35]; however, most disease is likely transmitted by younger, healthier individuals at low risk of severe outcomes. Inclusion of healthy children and working adults in influenza vaccination programs [36–38] could enhance the level of herd immunity and disrupt disease transmission, conferring significant indirect benefits.

In the modelling framework developed here, we considered the effect of several factors affecting VE, without explicitly encapsulating the underlying mechanisms that are also influenced by pathogen-host interactions. For example, the host conditions (e.g., age, co-morbid illness, and immunocompromised) are included as frailty of individuals [3, 4]. The model was parameterized with age-specific contact patterns of individuals in urban settings, which can affect the risk of infection per contact regardless of the level of vaccine-induced protection [21]. We did not explicitly include pre-existing immunity, as its level in different seasons is unknown and changes during each season due to various factors [7]. However, the effect of such immunity is to a large degree accounted for through interrelated parameters. First, while pre-existing cross-reactive immunity may not prevent infection, it can reduce the risk of developing clinical disease if infection occurred. This was taken into account by a parameter representing the probability of developing symptomatic infection. Second, any level of pre-existing immunity in the population reduces the severity and/or number of infections throughout the season. This is reflected in the attack rate, which varied in our model and calibrated to values within the estimated range for seasonal epidemics. We also considered different vaccine efficacies to account for changes in the virus characteristics that may lead to lower strain-specific protection levels. It is however important to distinguish between vaccine efficacy (prevention of illness among healthy vaccinated individuals enrolled in controlled clinical trials) and VE (prevention of illness in vaccinated populations). Unlike the former, the latter is subject to various factors in the real-world scenarios including repeated exposures, wider variation in response to vaccine, exposure to infection before an optimal immune response mounted upon vaccination, and variation in virus strains [7]. As we have shown, even a small antigenic distance in circulating strains (compared to vaccine composition) could lead to a significant reduction of VE. However, it is important to note that we have simplified the process by which influenza virus strains improve their antigenic distance. For example, some epitopes in the hemagglutinin-biding sites of the virus play a more dominant role than others and may have a high percentage of amino acid substitutions under strong immune pressure [39]. On the other hand, some substitutions occur rarely but may decrease the antigenic distance, thereby increasing the efficiency of mutated-virus neutralization by antibodies specific to the parent virus [40, 41]. Simplifying these mechanisms and using a sequence-based antigenic distance measurement [9], our model implements the effect of antigenic distance on vaccine efficacy linearly with the same probability of mutation at each site [39]. Extending the modelling framework proposed here with the inclusion of variabilities in amino acid substitutions and their effect on VE merits further investigation.

## Conclusions

Considering the qualitative aspects of our study, the results indicate that early vaccination remains an important public health strategy for alleviating the burden of seasonal influenza. Policy discussions on optimal timing of vaccination to reduce the effect of waning immunity will need to be considered in the context of unpreventable strain mutations during the epidemic. Potential solutions to limit the decline of VE may include an improved vaccination coverage of low-risk individuals, and a booster dose during the epidemic which represents a costly strategy and merits a rigorous evaluation for its cost-effectiveness.

## Supporting information

S1 Appendix Details of the model structure, parameterization and supporting results. (DOCX)
