# Supplementary material for: Projecting influenza vaccine effectiveness: A simulation study
Source: PLoS One. 2020 Nov 3;15(11):e0241549. doi: 10.1371/journal.pone.0241549 (PMC7608924; doi:10.1371/journal.pone.0241549)
Supplement: S1 Appendix — (DOCX) [file pone.0241549.s001.docx]

**S1 Appendix**

**Projecting influenza vaccine effectiveness: a simulation study**

**Thomas N. Vilches,^1^ Affan Shoukat,^2^ Claudia Pio Ferreira,^3^ Seyed M. Moghadas^4^**

^1^*Institute of Mathematics, Statistics and Scientific Computing, University of Campinas, Campinas SP, Brazil*

﻿^2^*Center for Infectious Disease Modeling and Analysis, School of Public Health, Yale University, CT, USA*

^3^*Institute of Biosciences, Department of Biostatistics, São Paulo State University, Botucatu SP, Brazil*

^4^*Agent-Based Modelling Laboratory, York University, Toronto, Ontario, Canada*

This supplementary information provides further details of the model structure, its parameterization, and additional results supporting the conclusions of the study.

**Disease dynamics**

The computational model was developed based on the epidemiological statuses of individuals, with infection dynamics represented in Figure S1.

**Figure S1.** Natural history and disease dynamics.

**Parametrization**

**Figure S2.** (a) Age distribution of the population [^[[1]](#endnote-1)^]; (b) simulated contact patterns; (c) frailty index as a function of age with segmented linear regression on the 2016 Canadian Community Health Survey data of chronic diseases [^[[2]](#endnote-2)^].

**Table S1**. Frailty index derived from the 2016 Canadian Community Health Survey data of chronic diseases [2]. The segmented linear regression on this data (Figure S2) were used to sample the age-dependent frailty index of individuals as described in the main text.

| **Age (years)** | 20 | 25 | 30 | 35 | 40 | 45 | 50 | 55 | 60 | 65 | 70 | 75 | 80 |
| --- | --- | --- | --- | --- | --- | --- | --- | --- | --- | --- | --- | --- | --- |
| **Frailty index** | 0.1755 | 0.1725 | 0.132 | 0.15 | 0.1875 | 0.2355 | 0.303 | 0.3975 | 0.4365 | 0.5145 | 0.6555 | 0.7155 | 0.6945 |

**Table S2.** Mean and standard deviation for the daily number of contacts in different age group [^[[3]](#endnote-3)^].

| Age | Mean number of daily contacts | Standard deviation |
| --- | --- | --- |
| 0-4 | 10.21 | 7.65 |
| 5-9 | 14.81 | 10.09 |
| 10-14 | 18.22 | 12.27 |
| 15-19 | 17.58 | 12.03 |
| 20-29 | 13.57 | 10.60 |
| 30-39 | 14.14 | 10.15 |
| 40-49 | 13.83 | 10.86 |
| 50-59 | 12.30 | 10.23 |
| 60-69 | 9.21 | 7.96 |
| 70+ | 6.89 | 5.83 |

**Table S3**. Vaccine coverage for aggregated age groups [^[[4]](#endnote-4)^].

| **Age** | **Vaccine coverage** | **95% confident interval** |
| --- | --- | --- |
| 6 months – 4 years | 26.5 | (20.1–32.9) |
| 5 – 12 years | 23.0 | (18.5–27.4) |
| 13 – 17 years | 23.2 | (17.0–29.4) |
| 18 – 49 years | 22.7 | (19.4–25.9) |
| 50 – 64 years | 38.2 | (34.2–42.3) |
| 65+ | 69.5 | (65.5–73.4) |

**Results**

***Temporal antigenic distance during the epidemic season due to strain mutations***

**Figure S3.** Temporal trends of maximum antigenic distance when the epidemic starts with only vaccine strains (d=0). Vaccine efficacy is 70% in a1-a6 and 30% in b1-b6. Attack rates are (a1,b1): 4%; (a2,b2): 8%; (a3,b3): 12%; (a4,b4): 20%; (a5,b5): 30%; (a6,b6): 40% (corresponding to model calibration in the absence of vaccination). Boxplots represent the variation in maximum antigenic distance with median shown by red circles.

**Figure S4.** Temporal trends of maximum antigenic distance when the epidemic starts with strains of positive antigenic distance in the range 0-0.04. Vaccine efficacy is 70% in a1-a6 and 30% in b1-b6. Attack rates are (a1,b1): 4%; (a2,b2): 8%; (a3,b3): 12%; (a4,b4): 20%; (a5,b5): 30%; (a6,b6): 40% (corresponding to model calibration in the absence of vaccination). Boxplots represent the variation in maximum antigenic distance with the median shown by red circles.

***Incidence of infection in the antigenic distance model***

**Figure S5.** Average incidence of infection among unvaccinated (a1-a6) and vaccinated (b1-b6) individuals in the antigenic distance model. Color curves correspond to epidemics starting with strains of different antigenic distance in the range 0-0.04. Vaccine efficacy was set to 70%. Attack rates are (a1,b1): 4%; (a2,b2): 8%; (a3,b3): 12%; (a4,b4): 20%; (a5,b5): 30%; (a6,b6): 40% (corresponding to model calibration in the absence of vaccination).

**Figure S6.** Average incidence of infection among unvaccinated (a1-a6) and vaccinated (b1-b6) individuals in the antigenic distance model. Color curves correspond to epidemics starting with strains of different antigenic distance in the range 0-0.04. Vaccine efficacy was set to 30%. Attack rates are (a1,b1): 4%; (a2,b2): 8%; (a3,b3): 12%; (a4,b4): 20%; (a5,b5): 30%; (a6,b6): 40% (corresponding to model calibration in the absence of vaccination).

***Incidence of infection in the waning immunity model***

**Figure S7.** Average incidence of infection among unvaccinated (a-c) and vaccinated (b-d) individuals in the waning immunity model. Color curves represent epidemics with different attack rates (corresponding to model calibration in the absence of vaccination). Vaccine efficacy was set to (a,b): 70%, and (c,d): 30%.

***Projected age-specific VE and reduction of illness in the antigenic distance model with 70% vaccine efficacy and 12% attack rate***

**Figure S8.** Projected vaccine effectiveness (a1-a5) and percentage reduction of illness (b1-b5) for different age groups in the antigenic distance model with the initial vaccine efficacy of 70%. The attack rate was calibrated for 12% (a moderate attack rate) in the absence of vaccination. Simulated scenarios correspond to the presence of circulating strains with different antigenic distances at the onset of epidemic. Boxplots represents the variation in projected VE and reduction of illness with median values shown by red circles.

***Projected age-specific VE and reduction of illness in the antigenic distance model with 30% vaccine and 12% attack rate***

**Figure S9.** Projected vaccine effectiveness (a1-a5) and percentage reduction of illness (b1-b5) for different age groups in the antigenic distance model with the initial vaccine efficacy of 30%. The attack rate was calibrated for 12% (a moderate attack rate) in the absence of vaccination. Simulated scenarios correspond to the presence of circulating strains with different antigenic distances at the onset of epidemic. Boxplots represents the variation in projected VE and reduction of illness with median values shown by red circles.

***Projected age-specific VE and reduction of illness in the waning immunity model with 12% attack rate and vaccine efficacies of 70% and 30%***

**Figure S10.** Projected vaccine effectiveness (a1,a2) and reduction of illness (b1,b2) in the waning immunity model with the initial vaccine efficacy of 70% and 30%. The attack rate was calibrated for 12% (a moderate attack rate) in the absence of vaccination. Boxplots represents the variation in projected VE and reduction of illness with median values shown by red circles.

**References**

1. . Government of Canada’s website available at http://www5.statcan.gc.ca/cansim/a26?lang=eng&retrLang=eng&id=0510001&&pattern=&stByVal=1&p1=1&p2=37&tabMode=dataTable&csid=, Accessed in February 2018. [↑](#endnote-ref-1)
2. . Government of Canada’s website available at http://infobase.phac-aspc.gc.ca:9600/PHAC/dimensionMembers.jsp?l=en&rep=i3212B12F133F4CE88AD13DB60CA37237&s#, Accessed 16 March 2018. [↑](#endnote-ref-2)
3. . Mossong J, Hens N, Jit M, Beutels P, Auranen K, Mikolajczyk R, Massari M, Salmaso S, Tomba GS, Wallinga J, Heijne J. Social contacts and mixing patterns relevant to the spread of infectious diseases. PLoS Med. 2008;5(3):e74. [↑](#endnote-ref-3)
4. . Public Health Agency of Canada (2018) 2016/17 Seasonal Influenza Vaccine Coverage in Canada. [↑](#endnote-ref-4)
